# Munificent Donation to the Medical College at Columbus, Ohio

**Published:** 1848-01

**Authors:** 


*Munificent donation to the Medical College at Columbus, Ohio.*—We have been favored with a copy of a printed deed of trust executed by Lyne Starling, of Columbus, Ohio, conveying the sum of thirty thousand dollars, "to aid in founding and sustaining a medical college in the city of Columbus," the amount to be paid in successive instalments within the next three years. It is directed in the deed that twenty thousand dollars be appropriated to the erection of a college edifice, and the remaining ten thousand to "sustain an infirmary or hospital, or some other benevolent institution under the supervision and control of said medical college, and in said city of Columbus,"

Seven Trustees are designated, vacancies to be filled by the board *in perpetuo.* Not less than too, nor more than three regular Physicians of Columbus, shall, at any time be members of the board of Trustees; and only two Professors, or officers of the College, shall, at any time be members of the board. The Institution is to incur no debts beyond their cash means for meeting them within one year. The Professors of the College to be appointed, and subject to be removed by the Trustees, and in the appointments, the Trustees are to look solely to the character and qualifications of candidates, and to the permanent interests of the Institution. The foregoing are the provisions of importance.

We have only time and space to express our admiration of the spirit and policy of this truly munificent donation, and to tender our unaffected congratulations to our confreres of the Columbus Institution.

